# Idiopathic Chylous Ascites in Pregnancy: A Case Report

**Published:** 2018-07

**Authors:** Bili ZHANG, Xueqin ZHANG, Yijing WANG

**Affiliations:** Dept. of Obstetrics, Xiamen Maternity and Child Health Care Hospital, Xiamen 361003, China

**Keywords:** Idiopathic chylous ascites, Pregnancy, Lymph

## Abstract

Idiopathic chylous ascites in pregnancy is extremely rare. Here, we report a 24-yr-old patient with idiopathic chylous ascites in pregnancy. The patient was hospitalized in Xiamen Maternity and Child Health Care Hospital, Xiamen China in 2014 due to G1P0 intrauterine pregnancy 39+2 week, LOA, and time of labor. The patient gave birth to a live baby boy (3.6 kg) by spontaneous vaginal delivery, with complete delivery of placenta. Three hours after delivery, the patient reported abdominal distension and pain, as well as asthma. Later, milky white liquid was drawn from left lower abdomen by puncture. Exploratory laparotomy was performed, and 800 ml milky white liquid was drawn from the abdominal cavity. Subsequently, drainage tube was placed in the abdominal cavity, and the abdomen was closed. After the surgery, the patient was given low-fat diet, supplemented with parenteral nutrition support and intravenous injection of antibiotics. Extubation was performed on day 3. On day 7 after surgery, the mother and baby left the hospital without any health problems. No abnormality was observed during six months of follow-ups. Idiopathic chylous ascites in pregnancy may be related to congenital lymphatic system dysplasia, and directly caused by chylous flow from ducts into abdominal cavity induced by progesterone during pregnancy or pressure from enlarged uterus during late pregnancy. After pregnancy, the disease is cured by the release of disease cause and the reduction of thoracic duct pressure.

## Introduction

Chylous ascites is milky white ascites formed by accumulated lipid-rich lymph liquid in the abdominal cavity, caused by trauma or lymphatic obstruction. Chylous ascites is clinically rare, with an incidence rate of 1/20000, but it can be caused by various kinds of diseases (1). The precise mechanism of idiopathic chylous ascites is still unknown. Possible causes include exudation of chyle from lymphatic ducts in the wall of the bowel or mesentery due to obstruction of normal lymphatic flow. The exudation of chyle may have been due to primary lymphatic dysplasia or other unknown causes (2). Idiopathic chylous ascites in pregnancy is extremely rare, with only two case reports being included in PubMed before Dec 2014 (1; 3).

Here, we report a case of idiopathic chylous ascites in pregnancy in Xiamen Maternity and Child Health Hospital.

## Case report

Prior written and informed consent were obtained from this patient and the study was approved by the ethics review board of Xiamen Maternity and Child Health Care Hospital. A 24 yr old pregnant woman with a height of 155 cm and a body weight of 64 kg was hospitalized in Xiamen Maternity and Child Health Care Hospital, Xiamen, China in 2014 (G1P0 intrauterine pregnancy 39+2 wk, LOA, and near the time of labor). Before hospitalization, no abnormal phenomenon was observed during prenatal follow-ups at hospital. After hospitalization, routine blood test, routine urine test, coagulation function, liver function, renal function, virus index, and infectious disease index were all normal. On the day of admission, the opening of cervix was 3 mm at 17:00, but the opening remained the same at 19:00. Due to cervical edema, the patient was administered with 10 mg diazepam by intravenous injection before rest. At 23:00, the cervix was fully open. At 23:45, the patient gave birth to a live baby boy (3.6 kg) by spontaneous vaginal delivery, with complete delivery of placenta. Three hours after delivery, the patient reported abdominal distension and pain, as well as asthma. Body examinations showed abdominal swelling, mild tenderness, no rebound pain, and positive shifting dullness. After that, milky white liquid was drawn from left lower abdomen by puncture.

At 3:50 on the next day, exploratory laparotomy was performed, and 800 ml milky white liquid was drawn from the abdominal cavity. Examination of the drainage fluid specimen showed the following characteristics of the fluid: fat globules under the microscope, a density of 1.021 g/ml, pH=7.40, blood cell count=3.5×10^9^/l, mainly lymphocytes, the concentration of triglyceride=1.28 mmol/l, and positive sultan fat staining. No obvious abnormalities in abdominal organs were found during the surgery. During 1 hour of observation in the surgery, no obvious liquid appeared in the abdominal cavity. Subsequently, drainage tube was placed in the abdominal cavity, and the abdomen was closed. After the surgery, the patient was given low-fat diet, supplemented with parenteral nutrition support and intravenous injection of antibiotics. During the first three days after surgery, the total peritoneal drainage fluid volume was 45 ml, and extubation was performed on day 3. Computed tomography scans of chest, abdomen, and pelvis were performed to exclude the possibility for tumor or other lesions. On day 7 after surgery, the mother and baby left the hospital without any health problems. No abnormality was observed during six months of follow-ups.

## Discussion

The cause of chylous ascites is not clear yet. It can be the result of various factors(3): i) abdominal malignant tumors may directly lead to lymphatic rupture by invasion or suppression; ii) infections may block lymph vessels, leading to vessel rupture and chylous ascites; iii) trauma such as abdominal surgery may damage lymph vessels or chylocyst; iv) congenital malformations in lymph vessel may lead to its dysplasia, stricture, and expansion; v) other diseases such as liver cirrhosis, nephrotic syndrome, or mesenteric lymphadenitis. Three mechanisms were proposed for the formation of chylous ascites: i) mesenteric base or chylocyst are obstructed, intestinal wall is dilated, and lymphatic vessels spontaneously rupture, followed by the leakage of chylus; ii) lymphatic vessels rupture after trauma or surgery, and chylus leaks via abdominal fistula of lymphatic vessels, accompanied by abnormal retroperitoneal lymphatic vessels; iii) chylus leaks via retroperitoneal giant lymphatic vessel wall (4). The laboratory examination characteristics of chylous ascites include (5): milky white in color, cell count between 232 and 2560/mm^3^, mainly lymphocytes, total protein concentration 14 – 64 g/l, normal sugar and amylase contents, low cholesterol level, and triglyceride concentration > 1.25 mmol/l. The level of triglyceride concentration is the most important index for the diagnosis of chylous ascites. Only two cases of idiopathic chylous ascites in pregnancy were reported according to PubMed. Both cases were found to have chylous ascites during caesarean section. Case 1 had a history of two deliveries, and the second time of delivery was caesarean section due to uterine inertia (6). No abnormality was found before the third delivery of case 1 (5). Case 2 had no history of delivery before but received caesarean section due to huge fetus (6). Before surgery, case 2 had obesity (body mass index, 38.9 kg/m^2^), congenital ventricular septal defect, congenital glaucoma, and thalassemia. Case 2 received routine pre-pregnancy examination and treatment, and the pregnancy was terminated on week 40 by caesarean section (6). For both cases, no abnormality was observed for abdominal organs, and no primary disease was found after surgery. Our patient, no abnormality was found during prenatal examinations. After normal vaginal delivery, abdominal distension, pain, and asthma appeared. The milky white liquid drawn from abdominal cavity was confirmed to be chylous ascites by subsequent laparotomy. No organic disease was observed during the laparotomy. The imaging data of a group of 40 cases were analyzed with chylous ascites and found that structural defects often occur in the lymphatic duct system, including lymph duct dilatation, lymphatic valvular function loss, chylous cyst, chylous stasis, obstruction or reflux (8). Lymphatic duct dysplasia was observed and significantly slow lymph flow in lymphatic radionuclide imaging of patients with spontaneous chylous ascites (2).

Case 1, case 2 and the case found in our hospital may be caused by the following reasons: i) congenital dysplasia of the lymphatic system, led to the dilated lymphatic duct in Case 1 (6); ii) effect of progesterone during pregnancy, led to the diastolic state of lymphatic duct; iii) enlarged uterus during late pregnancy, which may suppress the abdominal thoracic duct of the mother, and increase the pressure of thoracic duct. Together, these factors lead to the flow of chylous fluid from duct into abdominal cavity. The infiltrated chylous ascites has slow progression and long duration, and may only exhibit abdominal distension symptom. For case 2, chylous ascites was unexpectedly found during caesarean section, just because of the enlarged uterus during late pregnancy. For our patient, infiltration of chylous liquid may be aggravated by the pressure of thoracic duct during vaginal delivery. The infiltrated chylous liquid may further stimulate the peritoneum, caused abdominal distension, pain, and asthma in the patient three hours after the delivery.

## Conclusion

Idiopathic chylous ascites in pregnancy may be related to congenital lymphatic system dysplasia, and directly caused by chylous flow from ducts into abdominal cavity induced by progesterone during pregnancy or pressure from enlarged uterus during late pregnancy. After pregnancy, the disease is cured by the release of disease cause and the reduction of thoracic duct pressure.
